# Björk-Jarabak cephalometric analysis on CBCT synthesized cephalograms with different dentofacial sagittal skeletal patterns

**DOI:** 10.1590/2176-9451.19.6.046-053.oar

**Published:** 2014

**Authors:** Yalil Augusto Rodriguez-Cardenas, Luis Ernesto Arriola-Guillen, Carlos Flores-Mir

**Affiliations:** 1 Universidad Peruana Cayetano Heredia, Specialist in Orthodontics, National University of Colombia. Specialist in Oral and Maxillofacial Radiology, Universidad Peruana Cayetano Heredia; 2 Universidad Peruana Cayetano Heredia, Universidad Científica del Sur-UCSUR and Universidad Nacional Mayor de San Marcos, School of Dentistry, Department of Orthodontics, Associate Professor, Department of Orthodontics, School of Dentistry, Universidad Científica del Sur-UCSUR and Universidad Nacional Mayor de San Marcos, UNMSM; 3 University of Alberta, Department of Orthodontics, Associate Professor and Head of the Department of Orthodontics, University of Alberta

**Keywords:** Cephalometry, Computed tomography scanners, Malocclusion

## Abstract

**OBJECTIVE:**

The objective of this study was to evaluate the Björk and Jabarak cephalometric analysis generated from cone-beam computed tomography (CBCT) synthesized lateral cephalograms in adults with different sagittal skeletal patterns.

**METHODS:**

The sample consisted of 46 CBCT synthesized cephalograms obtained from patients between 16 and 40 years old. A Björk and Jarabak cephalometric analysis among different sagittal skeletal classes was performed. Analysis of variance (ANOVA), multiple range test of Tukey, Kruskal-Wallis test, and independent t-test were used as appropriate.

**RESULTS:**

In comparison to the standard values: Skeletal Class III had increased gonial and superior gonial angles (P < 0.001). This trend was also evident when sex was considered. For Class I males, the sella angle was decreased (P = 0.041), articular angle increased (P = 0.027) and gonial angle decreased (P = 0.002); whereas for Class III males, the gonial angle was increased (P = 0.012). For Class I females, the articular angle was increased (P = 0.029) and the gonial angle decreased (P = 0.004). Björk's sum and Björk and Jabarak polygon sum showed no significant differences. The facial biotype presented in the three sagittal classes was mainly hypodivergent and neutral.

**CONCLUSIONS:**

In this sample, skeletal Class III malocclusion was strongly differentiated from the other sagittal classes, specifically in the mandible, as calculated through Björk and Jarabak analysis.

## Abstract (pt)

**OBJETIVO:**

o objetivo deste estudo foi avaliar a análise cefalométrica de Björk-Jarabak realizada com base em telerradiografias sintetizadas a partir de tomografia computadorizada de feixe cônico (TCFC) em adultos com diferentes padrões esqueléticos sagitais.

**MÉTODOS:**

a amostra foi composta de 46 telerradiografias sintetizadas a partir de TCFC obtidas de pacientes entre 16 e 40 anos de idade. A análise cefalométrica de Björk-Jarabak foi conduzida entre as diferentes classes esqueléticas sagitais. Análise de variância (ANOVA), teste de Tukey, de Kruskal-Wallis e teste *t* independente também foram realizados.

**RESULTADOS:**

em comparação a valores padrão, a má oclusão de Classe III esquelética apresentava ângulos goníacos aumentados (p < 0,001). Essa tendência também foi evidente quando o sexo dos pacientes foi considerado. Pacientes Classe I do sexo masculino tiveram o ângulo sela diminuído (p = 0,041), o ângulo articular aumentado (p = 0,027) e o ângulo goníaco também diminuído (p = 0,002). Já os pacientes Classe III tiveram o ângulo goníaco aumentado (p = 0,012). Pacientes Classe I do sexo feminino tiveram o ângulo articular aumentado (p = 0,029) e o ângulo goníaco diminuído (p = 0,004). A soma de Björk e a soma dos polígonos de Björk-Jarabak não revelaram diferenças significativas. O biótipo facial presente nas três classes sagitais foi hipodivergente e neutro.

**CONCLUSÕES:**

na amostra do presente estudo, a má oclusão esquelética de Classe III foi fortemente diferenciada de outras classes sagitais, principalmente na mandíbula, conforme demonstrado pela análise de Björk-Jarabak.

## INTRODUCTION

The recent development of cone-beam computed tomography (CBCT) for craniofacial imaging has encouraged its use in Orthodontics by providing volumetric information that allows the development of three-dimensional models valuable for impacted teeth localization, TMJ visualization, among other applications.^1^^,^ 2 ^,^ 3 It has also allowed the production of 2D high resolution imaging without magnification.^4^ This latter aspect facilitates the use of CBCT synthesized cephalograms for orthodontic treatment planning. In this regard, three methods to simulate conventional two-dimensional cephalograms from CBCT images and volumetric data sets have been described:^5^ Lateral scout radiograph taken initially to confirm patient's positioning, maximum intensity projection (MIP), and ray-sum technique.

Replacement of conventional 2D radiographs by their 3D counterpart appears to be a trend.^7^ 3D volumetric imaging of the maxilla and mandible has been studied in various skeletal classifications.^8^ Moreover, no statistical differences between cephalometric analyses performed on conventional and CBCT-generated cephalograms of patients has been shown several times.^9^^,^ 11 ^,^ 12

CBCT-synthesized cephalograms have been used to perform cephalometric analyses, comparing the three types of 2D images that can be produced (conventional, ray-sum, or MIP).^9^ However, other authors have found better accuracy on ray-sum produced images, and reported it as a potential method to better simulate lateral cephalometric images from CBCT data sets.^10^ The latter is described in more detail in a previous study.^6^ In summary, the produced images can be thickened by increasing the number of adjacent voxels. This summation process is called "ray-sum", and can create an image that represents a specifically defined volume of the patient. By adding the intensity values of adjacent voxels along a particular section, its thickness increases and creates a thin plate of 5 to 10 mm, or thicker (more than 20 mm if desired), allowing the production of flat cephalograms without distortion, which can be exported and analyzed by means of cephalometric analysis programs.

Jarabak's cephalometric analysis,^13^ based on a study by Björk,^14^ has been used to compare facial variations of shape and size based on age, sex and race. Jarabak's cephalometric analysis mainly considers vertical intermaxillary relationships and uses the cranial base as reference. The final response of Jarabak's polygon to different sagittal skeletal malocclusions, including facial biotypes as shown in non-growing young adults, is an issue that has not been studied yet. The purpose of this study is, therefore, to evaluate Björk and Jabarak cephalometric analysis on CBCT-generated cephalograms with different dentofacial sagittal skeletal patterns.

## METHODS

The study was approved by local ethics committee. CBCT-synthesized lateral cephalograms from 46 subjects (24 men, 22 women) were randomly selected from an available database (Table 1). Sample size was calculated considering a gonial angle difference^15^ of 3° between Class I and III malocclusion as clinically relevant, with an expected variance of 9°. With a one-sided significance level of 0.05 and a power of 80%, a minimum of 12 patients per group was required.

**Table 1.** Descriptive statistics of the sample by skeletal class and sex.

Skeletal Class	Sex	n	ANB	FMA
Class I	Male	8	3.19	25.74
	Female	7	2.86	27.46
Class II	Male	8	7.12	31.01
	Female	7	7.84	32.00
Class III	Male	8	-4.04	32.02
	Female	8	-4.58	27.07
Total		46		

The final number of participants included was of 46 (15 for Class I, 15 for Class II, and 16 for Class III malocclusion subjects). Inclusion criteria were: CBCT with large field of view (FOV), and patients aged between 16 and 40 years (all subjects had complete craniofacial growth as determined by CVM 6).^16^ Participants were in centric occlusion (maximum intercuspidation) during CBCT imaging, and no chin positioner was used to avoid possible alterations in jaw position. Exclusion criteria were: Patients with severe asymmetries, known craniofacial syndromes, under active orthodontic treatment, with tooth loss (except for third molars) or with prior history of orthognatic surgery.

**Figure 1.**
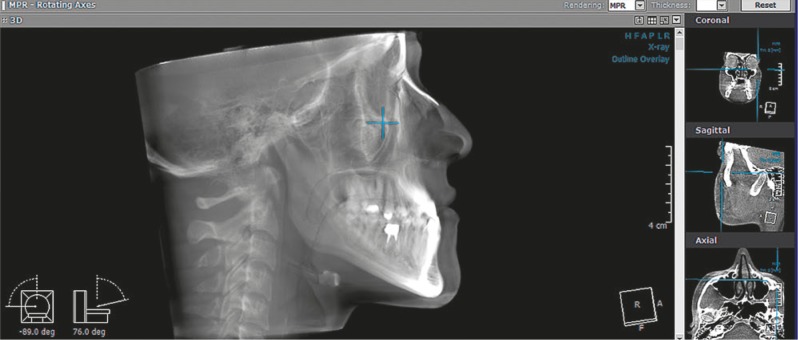
Example of CBCT cephalogram used in this study.

Imaging was performed with a Picasso Master 3D (Vatech, E-WOO Technology Co, Ltd, Republic of Korea). Device settings were set at 8 mA and 90 kV. Each ﬁeld of view mode was 20 cm X 19 cm. The image was processed with EZImplant 3D software which allowed the generation of ray-sum type generated 2D lateral skull projection cephalometrics (Fig 1).

## Cephalometric analysis

**Figure 2.**
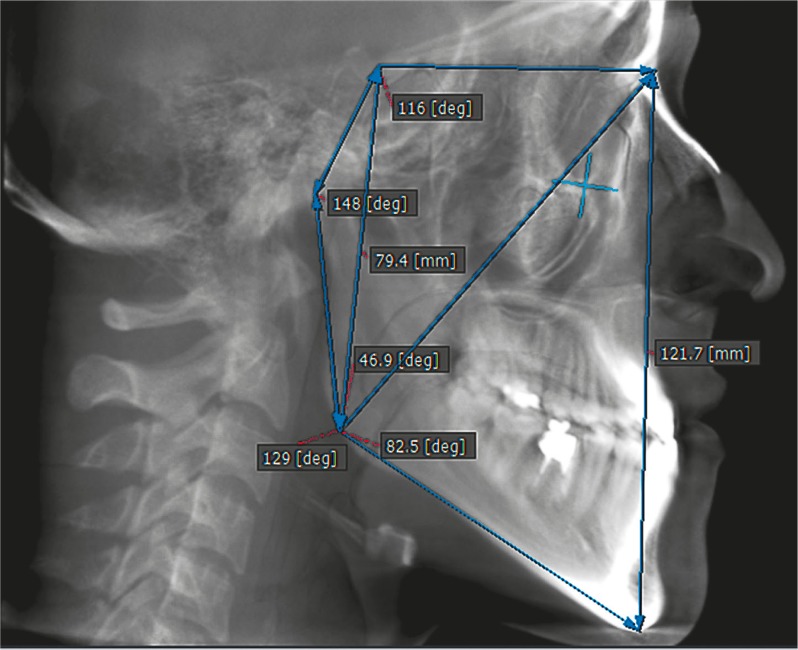
Angular and linear parameters for Björk and Jarabak analysis used in this study.

The cephalometric analysis was derived from Björk and Jarabak analysis^13^ (Fig 2) and included: N-S-Ar (saddle angle), S-Ar-Go (articular angle), Ar-Go-Me (gonial angle), Ar-Go-N (upper gonial angle), N-Go-Me (lower gonial angle) plus the following linear measurements: S-Go (posterior facial height), and N-Me (anterior facial height) (Fig 1).

Additionally, ANB angle was determined and analyzed for each participant. Participants were classified into three groups according to skeletal pattern: Skeletal Class I (0° ≤ ANB <4°), Class II (ANB ≥ 4°), and Class III (ANB < 0°). The definitions of points and angles used in this study were according to those described by Northway et al.^17^

## Methods for error analysis

Cephalometric tracings were performed by an orthodontist previously calibrated for the Björk and Jarabak analysis and with 10 years of experience drawing cephalograms. Intraexaminer reliability was assessed with the intraclass correlation coefficient (ICC) which gave a result greater than 0.90 for all measurements (confidence intervals between 0.900 - 0.999). In addition, Dahlberg error was less than 1° (0.25 to 0.99) in angular measurements and less than 0.8 mm (0.5 to 0.8) in linear measurements. All cephalometric tracings were drawn twice with a one-week interval in between.

## Statistical analysis

All statistical analyses were performed using SPSS v.19 for Windows (IBM SPSS, Chicago, Illinois, USA). Normal distribution was confirmed by Shapiro-Wilk tests. One-way analysis of variance (ANOVA) was performed to determine whether there were differences in angles across the sagittal malocclusion types, if normality and homogeneity of variance assumptions were satisfied; otherwise, the equivalent non-parametric Kruskal-Wallis test was used. In addition, analysis of variance (ANOVA) was performed to take into account the significant differences between males and females in terms of sagittal skeletal patterns. The post-hoc analysis was a Tukey HSD. For comparisons between sex, angular measurements and comparisons of Björk and Jarabak analysis, an independent t-test was used. Statistical significance was set at P < 0.05 for all tests.

## RESULTS

Descriptive statistics for ANB angle, FMA angle, sex and the number of patients for each sagittal skeletal class are shown in Table 1. Class III skeletal had increased gonial and superior gonial angles (P < 0.001) during intergroup analysis (Table 2).

**Table 2.** Evaluation of cephalometric Björk and Jarabak's measurements according to sagittal skeletal class.

Skeletal class	Cephalometric measurements	X	SD	Min	Max	S^2^	p (1,2,3)	Multiple comparison (p)
1. Class I	NSAr	122.27	6.32	114.00	134.00	39.92	0.07**	(I, III p = < 0.001) (II, III p = 0.018)*** (I, III p = 0.001) (II, III p = 0.009)***
	SArGo	148.27	8.81	132.00	161.00	77.64	0.054*	
	ArGoMe	122.87	4.05	117.00	129.00	16.41	0.001*
	ArGoN	43.32	7.84	30.70	57.60	61.49	0.001*
	MeGoN	76.48	4.89	65.20	83.30	23.87	0.124**
	NMe	118.85	6.99	106.90	132.90	48.91	0.928**
	SGo	80.99	5.47	72.70	91.50	29.97	0.152*
2. Class II	NSAr	122.73	7.51	102.00	135.00	56.35	
	SArGo	149.20	8.92	138.00	167.00	79.60	
	ArGoMe	125.93	6.15	116.00	139.00	37.78	
	ArGoN	44.73	5.71	34.90	53.20	32.56	
	MeGoN	79.32	6.49	72.60	91.30	42.08	
	NMe	119.39	6.74	105.70	127.90	45.42	
	SGo	78.35	4.59	72.10	88.40	21.10	
3. Class III	NSAr	121.63	6.02	113.00	133.00	36.25	
	SArGo	142.31	7.29	129.00	154.00	53.16	
	ArGoMe	132.81	8.89	119.00	146.00	79.10	
	ArGoN	51.32	3.49	43.30	56.60	12.20	
	MeGoN	81.89	6.20	74.00	92.50	38.47	
	NMe	120.09	9.36	109.20	135.00	87.55	
	SGo	76.84	7.18	66.00	88.90	51.54	

* ANOVA.

** Kruskal-Wallis.

*** Tukey.

In comparison to published standards:

For Class I males, the sella angle was decreased (P = 0.041), articular angle increased (P = 0.027) and gonial angle decreased (P = 0.002); whereas for Class III males, the gonial angle was increased (P = 0.012). For Class I females, the articular angle was increased (P = 0.029) and the gonial angle decreased (P = 0.004) (Table 3).

**Table 3.** Comparison between the studied sella, articular and gonial angles and the Björk and Jabarak standard by skeletal class and sex.

Skeletal class	Sex	Angle	SD	Standard	SD	p	Mean difference	95% confidence interval	
								Lower	Upper
Sella angle									
Class I	Male	118.50	4.50	123	5	0.041	-4.42	-8.9	-0.26
	Female	125.50	6.07	123	5	0.282	2.50	-2.58	7.58
Class II	Male	123.10	11.02	123	5	0.974	0.14	-10.05	10.34
	Female	122.37	2.87	123	5	0.558	-0.62	-3.03	1.78
Class III	Male	121.80	4.54	123	5	0.507	-1.12	-4.93	2.68
	Female	121.30	7.53	123	5	0.561	-1.62	-7.93	4.68
Articular angle									
Class I	Male	151.40	7.63	143	6	0.027	8.42	1.37	15.49
	Female	145.50	9.30	143	6	0.472	2.50	-5.28	10.28
Class II	Male	147.10	9.82	143	6	0.307	4.14	-4.94	13.23
	Female	151.00	8.28	143	6	0.029	8.00	1.08	14.92
Class III	Male	137.80	6.89	143	6	0.074	-5.12	-10.89	0.64
	Female	146.70	4.62	143	6	0.055	3.75	-0.11	7.61
Gonial angle									
Class I	Male	122.80	3.48	130	7	0.002	-7.14	-10.37	-3.92
	Female	122.80	4.73	130	7	0.004	-7.12	-11.08	-3.17
Class II	Male	125.80	7.69	130	7	0.204	-4.14	-11.26	2.97
	Female	126.00	4.98	130	7	0.058	-4.00	-8.17	0.17
Class III	Male	138.80	7.51	130	7	0.012	8.87	2.60	15.15
	Female	126.70	5.39	130	7	0.132	-3.25	-7.76	1.26

Independent t-test.

Björk's sum and Björk and Jabarak polygon sum showed no significant differences among the different sagittal patterns. (Table 4)

**Table 4.** Comparison between the sum (Björk) studied and the standard by skeletal class and sex.

Skeletal class	Sex	Sum (Björk) Studied	SD	Sum (Björk) Standard	SD	p	Mean difference	95% confidence interval	
								Lower	Upper
Class I	Male	392.70	5.20	396	6	0.074	-3.14	-6.70	0.42
	Female	393.80	6.70	396	6	0.263	-2.12	-6.26	2.01
Class II	Male	396.00	9.51	396	6	0.331	0.14	-6.05	6.33
	Female	399.00	5.37	396	6	0.957	3.37	-4.26	11.01
Class III	Male	398.40	4.80	396	6	0.274	2.62	-2.60	7.85
	Female	394.70	5.84	396	6	0.309	-1.12	-3.55	1.30

Independent t-test.

For this sample, the facial biotype presented in the three sagittal classes was mainly hypodivergent and neutral (Table 5).

**Table 5.** Characteristics of Björk and Jabarak facial height ratio by sex and skeletal class.

Class	Female	Facial type	Male	Facial type
I	67.30°	CCW — HIPO	69.04°	CCW—HIPO
II	64.62°	NEUTRO	66.74°	CCW— HIPO
III	63.62°	NEUTRO	64.28°	NEUTRO

CCW-HYPO: Counterclockwise rotation hypodivergent.

## DISCUSSION

Lateral cephalometric analyses have been extensively used to develop guidelines that aid in orthodontic diagnosis and treatment planning. CBCT images allow clinicians to reformat volumetric 3D data set to conventional 2D by simulating plane projections such as a synthesized lateral cephalometric view. Several studies have been conducted to assess the accuracy of cephalometric measurements using CBCT images;^18^^,^ 19 however, no previous study has analyzed Björk and Jarabak's cephalometric analysis in a young adult non-growing population. The sample of this study comprised non-growing patients, which was confirmed by Bacceti's analysis, revealing that all patients were on CS6. Thus, age was not a variable and there was no bias. Björk and Jabarak analysis provides extensive information about the facial biotype of a patient through only a few cephalometric measurements. Previous researchers have emphasized the need to expand available norms for adult populations.^20^^,^ 21 ^,^ 22

A few published studies have specifically used significant parts or all of Björk and Jabarak analysis. All of them were conducted only on growing individuals with different facial biotypes. Chung et al^23^ reported the longitudinal craniofacial growth changes in untreated skeletal Class I subjects with low, average, and high MP-SN angles. They found that the SNA and SNB angles increased with age in all groups. Moreover, Alexander et al^24^ reported cephalometric growth changes in untreated Class III malocclusions by using semi-longitudinal cephalometric records. They found that the length of the anterior skull base increases with age less than 1 mm per year for women and around 1 mm for men. This increase is similar to Class I subjects; however, the longitudinal nature of this study is not accurate, because the sample comparisons between age groups were not performed on the same initial study group.

Reyes et al^25^ provided an estimate of facial growth in Class III malocclusion and found that the sella angle is smaller in Class III than in subjects with normal occlusion in both males and females. A report by Kuramae et al^26^ found that cephalometric measurements calculated for black Brazilian patients were similar to Jarabak's standards, except for S-N mean value for female patients, which was significantly lower than the established Jarabak's standard. The application of Björk and Jarabak analysis in all mentioned studies reinforces its relevance in the current context of orthodontic diagnosis, but no reports highlight findings on an adult population.

Because subjects at the same chronological age may have different skeletal maturation levels, evaluation of non-growing subjects may be important to determine specific characteristics of a given skeletal class. Dibbets^27^ stated that differences in mandibular size between Angle classes emerge later during development, and therefore, these differences are more likely to be found in adult samples. Kerr and Hirst,^28^ in a longitudinal study, found that the craniofacial characteristics of subjects with normal and postnormal occlusions became more defined with advancing age. These studies evaluated growing subjects at various ages, but none considered non-growing adults.

Regarding the present results, the significant differences found for sex in Class I malocclusion cases correspond to the sella angle. The behavior of this angle is strongly linked to the behavior of facial height. If the angle is small, the condyle is projected downward and slightly forward reflecting an increase in posterior and anterior facial height. This same characteristic was also observed for facial height on the skeletal Class III group. This result is consistent with the findings by Baccetti et al^29^ who studied a population between 3 and 57 years old. They found that Class III malocclusion is associated with a significant degree of sexual dimorphism in craniofacial parameters, especially from the age of 13 onward. In women, the sella angle turned out to be broader than in men, causing backward projection of the condyle and generating a slightly retrognathic profile reflected in the associated convex facial pattern. This sex characteristic was also reported by Pecora et al.^21^ The significant differences found in our study according to sex in Class III patients on the articular angle level do not coincide with the findings by Baccetti et al^29^ who found no sex differences at this angle, the so-called "cranial bending angle", neither sex differences in Class II. This result is consistent with findings by Chung and Wong^30^ who studied Class II growing patients and found that skeletal changes in angular measurements were similar in male and female groups. However, linear measurements showed significant differences. Our results suggest that the behavior of the saddle angle affects facial height, and it is also reflected in the dentofacial skeletal pattern.

In our study, the facial growth pattern of Class III patients was strongly differentiated from other skeletal classes. There were significant differences found in the gonial and superior gonial angles on Class III subjects compared with Class I and II subjects. The development of Class III is multifactorial and complex, being derived from different combinations of dental and skeletal factors, changes in magnitude, direction, and timing of craniofacial growth. The findings of this study indicate a specific characterization of adult Class III subjects in which the behavior of their facial growth tends to be hyperdivergent, derived from the opening of the gonial angle and upper gonial angle and the projection of the symphysis of the chin forward. Other known factors that contribute to this condition are size, position and shape of the maxilla, mandible, skull base, teeth and glenoid fossa.

The sum of the sella, articular, and gonial angles according to Björk is one of the parameters that define the type of growth in a subject. The estimated value is 396 ± 6°, for an individual with neutral growth.^31^ Variations on this estimate can cause hyper or hypodivergent facial growth tendencies. In our study, the behavior of the polygon sum (Björk) showed no significant differences in relation to the published standard for the three sagittal skeletal classes. This reveals that despite differences between the various angles between skeletal classes, the result of growth was similar in this sample. In addition, an increased angle in one sagittal skeletal class can be compensated with the decrease of another angle on the same group. Saltaji et al^32^ evaluated the association between vertical facial morphology and overjet in untreated Class II subjects. They performed an analysis of the performance criteria of the sum (Björk) and facial proportions, and found a strong relationship between overjet, the sum (Björk), gonial angle and lower gonial angle. Their findings are in agreement with our results. Furthermore, findings such as the behavior of the lower gonial angle in Class III subjects are interesting. This angle has a strong tendency to increase in this sagittal skeletal pattern with respect to the other sagittal skeletal classes. This difference reinforces the vertical and hyperdivergent pattern of this sagittal skeletal class.

Also, similar findings were reported for the sella angle when skeletal Class I and Class III were compared in men. This angle is also called "angle of the cranial base" and there is no consensus if the articular or the basion point should be used. Proff et al^33^ found a statistically significant reduction in this angle, with a mean of 17.7 ± 3.05^o^ in Class III subjects. Guyer et al^34^ also reported an acute cranial base angle compared with skeletal Class I subjects in subjects growing up to 15 years. In the present study, there were almost no changes in this angle. No statistically significant differences were found, with skeletal Class III patients reporting the lowest values. The role of the cranial base is still controversial, and some authors argue that the cranial base in skeletal Class III subjects did not differ morphologically from the one in a Class I normal profile.^35^

The present study can shed some additional light on our understanding on how the sum of Björk and Jarabak's polygon behaves in different sagittal skeletal relations. According to the proportion calculation between the posterior facial height (S-Go) and the anterior facial height (N-Me), one subject can be considered hyperdivergent if this ratio is 59% or less, hypodivergent if it is 65% or more and neutral if proportion is between 60 to 64%.^13^ Our sample of Class I male and female adults had an hypodivergent biotype. Class II women had a neutral biotype, while Class II men were hypodivergent. In Class III, there were no sex differences and the facial biotype was neutral. Therefore, in our sample, there was a tendency to develop hypodivergent growth pattern in the three sagittal skeletal classes with a mandibular rotation in counterclockwise direction. According to these findings, the study population can be characterized not only by the three facial biotypes defined by Björk, since almost all of them are classified as hypodivergent or neutral facial growth, and the most extreme vertical cases were classified as neutral. In this regard, the verticality criteria in the facial subject context can be highlighted. Also, this issue is not considered by the ANB in the classic sagittal skeletal classification.

The deep vertical orientation described on Class III subjects was demonstrated with the gonial angles in a downward and backward direction on Björk and Jabarak analysis. This finding may be of paramount importance as it adds clinical information to other studies on Class III patients.

## CONCLUSIONS

Björk and Jabarak cephalometric analysis on CBCT synthesized cephalograms with different dentofacial sagittal skeletal patterns showed a downward and backward direction at the gonial and superior gonial angle on Class III sagittal malocclusion subjects.
